# Shellfish as a Potential Source of Hepatitis E Virus: Epidemiological Evidence, Biological Plausibility, and Research Gaps

**DOI:** 10.3390/v18020220

**Published:** 2026-02-09

**Authors:** Hiroaki Okamoto

**Affiliations:** Division of Virology, Department of Infection and Immunity, Jichi Medical University School of Medicine, 3311-1 Yakushiji, Shimotsuke 329-0498, Tochigi, Japan; hokamoto@jichi.ac.jp; Tel.: +81-285-58-7404; Fax: +81-285-44-1557

**Keywords:** hepatitis E virus (HEV), foodborne, shellfish, transmission, epidemiology, surveillance

## Abstract

Hepatitis E virus (HEV) is an important cause of acute and chronic hepatitis worldwide, transmitted primarily through waterborne exposure and zoonotic foodborne pathways. In recent years, shellfish have attracted growing attention as a potential vehicle for HEV transmission. This interest is driven by epidemiological observations linking shellfish consumption to human HEV infection and by repeated detection of HEV RNA in bivalve mollusks across multiple geographic regions. This review critically evaluates the current evidence by integrating epidemiological data, environmental and food surveillance studies, and mechanistic insights into viral accumulation in shellfish. Signals from outbreak investigations, observational studies, seroepidemiological surveys, and case reports suggest that shellfish may contribute to HEV exposure. However, these findings are largely associative, methodologically heterogeneous, and limited by the absence of explicit documentation of raw or undercooked shellfish consumption in many cases. To date, no study has recovered infectious HEV from shellfish, nor has any established molecular epidemiological linkage between shellfish-derived HEV and human infections. Mechanistic knowledge from norovirus and hepatitis A virus demonstrates that bivalves can bioaccumulate enteric viruses through filter feeding, yet HEV-specific processes governing viral binding, persistence, and infectivity within shellfish remain poorly defined. Surveillance data reveal marked geographic variation in HEV RNA detection among shellfish species and production areas. Overall, existing evidence supports shellfish as a biologically plausible but unconfirmed source of HEV exposure. Addressing key knowledge gaps—particularly through direct infectivity assessments and high-resolution molecular linkage studies—will be essential to determine the public health significance of shellfish within the broader ecology of HEV transmission.

## 1. Introduction

Hepatitis E virus (HEV) is a major cause of acute viral hepatitis worldwide and represents a heterogeneous public health challenge with substantial geographic variability [1,2]. Globally, the burden of HEV infection is considerable: an estimated 20 million infections and more than 3 million symptomatic cases occur each year, predominantly in low- and middle-income countries where waterborne transmission remains endemic [3,4]. In contrast, in industrialized regions, HEV is increasingly recognized as a zoonotic and foodborne pathogen, with most autochthonous cases arising from sporadic exposure rather than large outbreaks linked to contaminated water sources [5,6,7]. In immunocompromised individuals—including solid-organ transplant recipients, patients with hematologic malignancies, and people living with the human immunodeficiency virus—persistent HEV infection can occur, potentially leading to chronic hepatitis and cirrhosis [8,9,10]. Liver failure after HEV infection occurs in a subset of patients, including pregnant women, older men, those with underlying chronic liver disease, and immunocompromised patients [11,12]. Additional extrahepatic manifestations, particularly neurological and renal complications, further contribute to HEV’s clinical spectrum [10,13].

HEV exists in two particle forms: the non-enveloped, membrane-unassociated form (neHEV), found in bile and feces, and the quasi-enveloped form (eHEV), which circulates in blood and is released from infected cells into culture supernatants [14,15,16,17]. HEV is a small, positive-sense, single-stranded RNA virus classified within the family *Hepeviridae*, subfamily *Orthohepevirinae*, species *Paslahepevirus balayani* [18]. Its ~7.2 kb genome contains three major open reading frames (ORF1–3), encoding a nonstructural polyprotein involved in replication, a capsid protein, and a multifunctional phosphoprotein, respectively [18]. Despite its simple genomic organization, HEV displays extensive genetic diversity, currently defined in at least eight genotypes (HEV-1 to HEV-8), each with distinct ecological and epidemiological characteristics [18].

HEV-1 and HEV-2 infect only humans and are transmitted primarily via contaminated drinking water, frequently causing large outbreaks in regions with inadequate sanitation. In contrast, HEV-3 and HEV-4 have a broad animal host range—including domestic pigs, wild boars, deer, and rabbits—and are responsible for most sporadic and foodborne infections in Europe, North America, and East Asia [5]. Domestic pigs, wild boars, and deer are the primary animal reservoirs of HEV, and numerous strains from other animal species have demonstrated the capacity to cross species barriers and infect humans [19]. Transmission typically occurs through consumption of raw or undercooked animal-derived products, especially pork and wild game meat, although additional exposure pathways have increasingly been considered [20].

Beyond classical foodborne transmission via meat, HEV RNA is frequently detected in environmental matrices such as surface water, wastewater, and sewage, suggesting that aquatic environments may contribute to viral dissemination [21,22,23]. These observations have stimulated interest in water-associated foods as potential vehicles for HEV. In particular, shellfish—especially bivalve mollusks such as oysters, mussels, and clams—have emerged as a plausible source of exposure.

Bivalve shellfish are well known to bioaccumulate enteric viruses through filter feeding, and their role in transmitting non-enveloped RNA viruses such as norovirus and hepatitis A virus (HAV) is firmly established [24,25,26,27,28,29,30]. Over the past decade, increasing epidemiological and environmental evidence has suggested that shellfish may also contribute to HEV exposure. Such evidence includes case reports implicating shellfish consumption as well as numerous surveillance studies detecting HEV RNA in shellfish harvested from coastal and estuarine environments across multiple countries [31,32,33,34].

Nevertheless, the role of shellfish in HEV transmission remains incompletely understood. Critical uncertainties persist regarding the biological plausibility, infectivity, and actual public health significance of HEV detected in shellfish. To date, direct confirmation of shellfish-borne HEV infection—supported by molecular epidemiological linkage—is limited, and the extent to which HEV RNA positivity reflects true infection risk remains debated.

This review synthesizes current evidence regarding shellfish as a potential source of HEV exposure. Epidemiological evidence from human infection cases suggesting an association between shellfish consumption and HEV exposure is examined, followed by a discussion of the biological mechanisms underlying viral accumulation in shellfish and the infectivity of accumulated viruses. Country- and species-specific surveillance data on HEV RNA detection in shellfish are then reviewed, and preventive measures, knowledge gaps, and future research priorities are addressed.

## 2. Why Shellfish Are Considered as a Potential Source of HEV Infection: Epidemiological Evidence from Human Cases

Epidemiological interest in shellfish as a potential source of HEV exposure has arisen from a small but recurring set of observations in which shellfish consumption was reported more frequently among HEV cases than among non-cases, or was otherwise highlighted during outbreak investigations. Importantly, the strength of evidence varies substantially across study designs—from hypothesis-generating case reports to analytic epidemiological studies—and, in most instances, shellfish has been implicated through association rather than direct virological confirmation of the suspected food item (Table 1) [35,36,37,38,39,40].

### 2.1. Outbreak and Cluster Investigations Suggesting an Association with Shellfish Consumption

The most widely cited example is the hepatitis E outbreak detected among passengers returning from a world cruise [35]. In this investigation, acute infection was identified serologically among a subset of passengers, and sequencing of viral RNA from several case-patients indicated HEV-3, closely related to European strains. In the risk factor analysis, shellfish consumption on board was significantly associated with acute HEV infection (odds ratio 4.27; 95% CI 1.23–26.94), leading the authors to propose a probable common-source foodborne outbreak. Nevertheless, the suspected food vehicle could not be confirmed microbiologically because the implicated shellfish (or retained portions thereof) were not available for virological testing, and thus the inference rests on epidemiological association and plausibility rather than molecular linkage between a food sample and human cases.

### 2.2. Observational Studies in Endemic/Industrialized Settings Where Shellfish Appears as a Reported Exposure

Beyond outbreak investigations, shellfish has repeatedly appeared as a candidate exposure in observational studies of acute hepatitis E. In a national survey of acute hepatitis E in France, the authors reported that, among indigenous metropolitan cases, the most relevant and/or frequent possible risk factors included drinking water from a personal supply and uncooked shellfish consumption, together with other exposures such as contact with pigs [36]. As in many questionnaire-based investigations, these data support shellfish as a plausible exposure but do not establish causality, particularly in settings where multiple food and environmental exposures co-occur. 

However, not all analytic studies identify shellfish as a risk factor. For example, a more recent case–control study conducted in the Netherlands (2015–2017) reported that consumption of shellfish was not identified as a risk factor for acute hepatitis E [37]. Such findings underscore that the contribution of shellfish to HEV exposure may be geographically dependent and context-dependent, and that associations may be sensitive to background dietary patterns, the dominant HEV genotype circulating in humans and animals, and differences in sanitation infrastructure and coastal contamination pressures.

### 2.3. Case Reports and Exposure Histories Consistent with, but Not Proving, Shellfish-Associated HEV Infection

Several case reports have described acute hepatitis E following the consumption of raw or undercooked shellfish, with exposure timelines consistent with disease onset [41,42,43,44,45,46]. One illustrative case involved a Japanese traveler who developed hepatitis E after returning from Vietnam; ingestion of uncooked shellfish was reported prior to symptom onset, and the infecting HEV-4 strain was genetically close to a Vietnamese isolate. Importantly, his wife and daughter, who accompanied him but did not consume raw shellfish, remained negative for anti-HEV IgM/IgG and HEV RNA throughout his illness [38]. While such reports are valuable for hypothesis generation—particularly when travel-associated exposure is plausible—they provide limited evidence to conclusively attribute infection to shellfish, as alternative sources of exposure (such as contaminated water or other foods) often cannot be fully excluded in retrospective clinical histories.

### 2.4. Interpreting the Epidemiological Signal: What Is Established and What Remains Uncertain

Taken together, the epidemiological literature provides recurrent signals of association between shellfish consumption and HEV infection in specific contexts, including at least one large outbreak investigation [35] and several observational studies [39,40]. At the same time, the evidence base remains limited by several constraints common to foodborne viral epidemiology: reliance on self-reported dietary histories, co-exposures to better-established vehicles (notably pork and game products for zoonotic genotypes), and the frequent absence of retained food specimens for direct viral detection and sequencing. Moreover, the existence of well-conducted studies in which shellfish was not identified as a risk factor suggests that any shellfish contribution may be heterogeneous, potentially reflecting local contamination dynamics and differences in shellfish species, harvesting areas, and culinary practices [37].

These uncertainties motivate the need to consider whether shellfish exposure is biologically plausible for HEV and under what conditions it could translate into infection risk. Accordingly, the next section discusses the mechanisms by which bivalve shellfish can concentrate enteric viruses through filter-feeding and summarizes what is known about the infectivity and stability of viruses accumulated in shellfish tissues, drawing lessons from norovirus and HAV while highlighting what remains unknown for HEV.

## 3. Filter Feeding, Viral Bioaccumulation, and Infectivity of Viruses Concentrated in Shellfish

Bivalve shellfish, including oysters, mussels, and clams, are suspension feeders that continuously filter large volumes of surrounding water to extract organic particles [47]. During this process, microorganisms present in the water column—including bacteria and viruses—can be efficiently retained and concentrated within shellfish tissues, particularly in the digestive gland [48]. This biological characteristic underlies the long-recognized role of shellfish as vehicles for human exposure to enteric viruses and forms the mechanistic basis for considering shellfish as potential sources of foodborne viral infections [49].

### 3.1. Mechanisms of Viral Accumulation in Bivalve Shellfish

Viral accumulation in shellfish is not a passive process but involves a combination of physical filtration and specific interactions between viral particles and host tissues. Studies using human norovirus and HAV have demonstrated that viral particles can bind to carbohydrate ligands, such as histo-blood group antigen (HBGA)-like molecules, expressed on the epithelial surfaces of the digestive tissues of oysters and other bivalves [50,51,52]. These interactions can promote selective retention and prolonged persistence of viruses within shellfish, even after environmental virus concentrations decline [53].

In addition, viral accumulation efficiency is influenced by environmental factors, including water temperature, salinity, turbidity, and the degree of fecal contamination from human or animal sources. Seasonal variation in viral detection rates in shellfish has been reported for norovirus and HAV, reflecting both changes in environmental loading and shellfish physiology [54]. Importantly, depuration procedures commonly used in the shellfish industry to reduce bacterial contamination are largely ineffective against viruses, which may remain detectable in shellfish tissues despite prolonged purification in clean seawater [55,56].

### 3.2. Infectivity of Viruses Accumulated in Shellfish: Lessons from Norovirus and HAV

For norovirus and HAV, the public health relevance of shellfish contamination is well established. Numerous outbreaks of acute gastroenteritis and hepatitis A have been conclusively linked to the consumption of raw or undercooked shellfish, supported by epidemiological investigations and, in some cases, molecular matching between patient isolates and viruses detected in shellfish samples [27,57,58]. Experimental studies have further demonstrated that both norovirus surrogates and HAV can retain infectivity after accumulation in shellfish tissues and can persist for extended periods under refrigeration or typical storage conditions [59,60].

Notably, current evidence indicates that HAV, similar to HEV, exists in two distinct particle forms: a quasi-enveloped form, which is cloaked in host-derived membranes and circulates in the bloodstream [16,61], and a non-enveloped form excreted in feces after bile acids remove the envelope during biliary transit [16,61]. The non-enveloped HAV virions exhibit remarkable environmental stability—resistant to heat, acidic conditions, and desiccation—enabling prolonged infectivity in shellfish and other food matrices [62]. In contrast, norovirus, historically regarded as unculturable, has recently been propagated in human intestinal enteroid and organoid (HIE/HIO) systems [63]. Although direct studies on norovirus environmental stability remain limited, persistence of infectious norovirus has been demonstrated for several weeks in estuarine water—conditions analogous to shellfish-growing environments [64]. Additionally, outbreak investigations and human challenge studies consistently document norovirus’s high infectivity and resistance in food and water, particularly via bivalve shellfish accumulation of virus from contaminated waters [49]. Together, these data provide a compelling biological rationale for shellfish-mediated transmission of both HAV and norovirus, based on their form-specific stability traits and demonstrated infectivity under relevant environmental conditions.

### 3.3. Implications for HEV: What Is Known and What Remains Uncertain

Compared with norovirus and HAV, the public health relevance of HEV accumulation in shellfish remains far less defined. Like other enteric viruses, HEV is shed into the environment in a non-enveloped form, sharing similar physicochemical characteristics that support environmental stability [65]. HEV RNA has been repeatedly detected in surface waters, wastewater, and sewage, indicating that aquatic environments can serve as reservoirs or conduits for viral dissemination [21,22]. Only limited studies have progressed beyond RNA detection to demonstrate the presence of infectious HEV in water samples using cell culture systems. Notably, Salvador et al. [66] reported the recovery of infectious HEV from environmental water samples using Vero E6 cell cultures. These findings support the plausibility that HEV present in contaminated waters could be taken up and concentrated by filter-feeding shellfish.

However, critical differences exist. Unlike HAV, for which shellfish-borne transmission has been firmly established [29,30], direct evidence that HEV accumulated in shellfish remains infectious to humans is lacking. To date, most shellfish-related HEV studies rely on molecular detection of viral RNA, without demonstration of infectivity using cell culture systems or animal models. Furthermore, the specific interactions between HEV particles and shellfish tissues—such as receptor-mediated binding analogous to HBGA interactions described for norovirus—have not been elucidated.

Another unresolved issue concerns the relative contribution of human versus animal sources of HEV contamination in aquatic environments. HEV-1 and HEV-2 infect only humans and are frequently implicated in large waterborne outbreaks via human fecal contamination, particularly in regions with poor sanitation [67]. Meanwhile, zoonotic genotypes HEV-3 and HEV-4 are prevalent in domestic pigs and wild animals; domestic pigs shed large quantities of HEV-3 (around 10^5^ genome copies per gram of feces), representing a major source of environmental contamination via farm waste and slaughterhouse effluents [68,69]. Agricultural runoff and slaughterhouse effluents have thus been proposed as important contributors to environmental HEV-3/4 loading [69]. It remains unclear whether shellfish preferentially bioaccumulate HEV from human or animal origins; filter-feeding bivalves have been shown to concentrate HEV-3 preferentially in their digestive tissues [70]. Furthermore, potential genotype-specific differences in environmental stability have been hypothesized: zoonotic HEV-3 subtypes exhibit higher genomic variability and may possess greater persistence in aquatic environments compared to the more conserved human-restricted HEV-1/2 [71]. These differences suggest that genotype-specific stability or accumulation efficiency could influence the persistence of HEV in aquatic settings, but further targeted research is necessary.

### 3.4. Bridging Epidemiology and Surveillance

Taken together, existing knowledge from norovirus and HAV provides a compelling framework for understanding how shellfish could act as vehicles for HEV exposure. At the same time, the absence of direct infectivity data and molecular epidemiological linkage between shellfish-derived HEV and human cases necessitates cautious interpretation of available findings. This gap underscores the importance of systematic surveillance studies that quantify HEV RNA in shellfish across different geographic regions and species, while also highlighting the need for methodological standardization and complementary approaches to assess public health relevance.

The following section reviews country- and species-specific surveys of HEV RNA detection in shellfish, focusing on prevalence, viral loads, genotypes, and methodological considerations that shape current understanding of shellfish-associated HEV contamination.

## 4. Detection of HEV RNA in Shellfish: Country- and Species-Specific Surveillance Studies

Following epidemiological observations suggesting a possible association between shellfish consumption and HEV infection, numerous surveillance studies have examined the presence of HEV RNA in bivalve mollusks collected from coastal and estuarine environments [19]. These studies, conducted across different geographic regions and shellfish species, provide valuable insights into environmental contamination and potential exposure pathways. However, reported prevalence and viral loads vary substantially, reflecting heterogeneity in sampling strategies, analytical methods, environmental conditions, and contamination sources. This section summarizes surveillance findings by geographic region, emphasizing studies in which methodology and prevalence are clearly described (Table 2) [31,32,33,34,70,72,73,74,75,76,77,78,79,80,81,82,83,84,85].

### 4.1. Europe

Europe has generated the most extensive body of surveillance data on HEV in shellfish, mirroring the region’s high incidence of autochthonous HEV-3 infections in humans. One of the earliest reports came from Scotland, where HEV RNA was detected at unexpectedly high frequency (85%, 41/48) in wild mussels (*Mytilus* spp.) collected from intertidal zones [31]. Viral loads reached several log_10_ IU/mL in tissue homogenates, and phylogenetic analysis identified HEV-3 strains closely related to those circulating in European humans and pigs, suggesting contamination from land-based sources.

Subsequent investigations in the United Kingdom extended surveillance to retail shellfish. A large survey of mussels and oysters purchased from Scottish supermarkets detected HEV RNA in 2.9% (9/310) of samples, generally at low concentrations near the assay limit of detection [32]. Genotyping confirmed HEV-3, and sample processing followed ISO/TS 15216-aligned procedures [88], enhancing comparability with surveillance data for HEV in other regions.

In Southern European, HEV RNA has been repeatedly detected in shellfish harvested from aquaculture-intensive regions. Several surveys from Galicia, Spain—a major shellfish-producing area—reported HEV RNA in mussels, clams, and cockles collected from coastal rías [33,72]. In an 18-month longitudinal study, 24.4%, 41/168) of samples tested positive, with viral loads ranging from below quantification limits to more than 10^5^ genome copies per gram of digestive tissue. Sequencing revealed HEV-3 strains clustering with locally circulating swine-derived HEV, supporting the hypothesis of zoonotic environmental contamination [33].

In Italy, findings have been more variable [73,74]. Surveys in shellfish production and retail settings generally report low detection frequencies (<3%), with sporadic identification of HEV-3. An investigation of polluted sites in Tuscany reported HEV-1 at 8.4% (3/37), detected alongside other enteric viruses [75]. Conversely, several studies—conducted in production areas in Italy (e.g., Gulf of Naples) [76], Denmark [77], France [70], and Germany [78]—reported no detectable HEV RNA, although sample sizes varied. Supporting this geographic variability, HEV RNA was detected in commercial mussels from Spain (6%, 3/51) but not in mussels from Finland (0/51) within the same study [79].

### 4.2. Asia

Although fewer systematic surveys have been conducted in Asia—despite a high overall burden of HEV infection—the available data indicate that shellfish contamination with HEV RNA is not confined to Europe.

In China, multiple studies have examined shellfish harvested from coastal and estuarine areas influenced by river discharge and intensive pig farming [34]. Along the Bohai Gulf, HEV RNA was detected in approximately 15–20% of samples, including ark shells (*Scapharca subcrenata*), blood clams (*Anadara granosa*), and Manila clams (*Ruditapes philippinarum*). Detected strains were predominantly HEV-4, consistent with circulating genotype in humans and pigs [34]. Additional surveys in Hebei Province reported prevalence estimates of 5.0% (12/240) and 9.6% (34/354) [80,81].

In South Korea, HEV RNA has been detected in raw oysters intended for consumption, with reported prevalence of 8.7% (14/161) [82].

In Japan, detection of HEV RNA in freshwater bivalves collected from river systems suggests that shellfish can serve as indicators of HEV contamination in aquatic environments [83]. A study from Hokkaido found no HEV RNA in oysters (0%, 0/517), though HEV-3 RNA was detected in one of 37 seawater samples (20 L each), indicating marine contamination despite negative shellfish findings [84].

In Thailand, a survey of 213 edible bivalve from aquaculture and retail markets detected HAV RNA in 3.8% of samples but no HEV RNA [85].

### 4.3. Africa

Although reports of HEV contamination in African shellfish remain scarce, HEV contamination of shellfish appears rare in African surveillance studies. In Morocco, oysters and clams collected from the Oualidia Lagoon (*n* = 104) were tested using ISO/TS 15216–based RT-PCR [88], with no HEV RNA detected [86]. In Egypt, HAV, norovirus, and rotavirus were detected in market-purchased shellfish [89]. Broader reviews of foodborne and waterborne viruses in Africa highlight widespread environmental contamination pressures but confirm that empirical detection of HEV in shellfish remains extremely limited [90,91].

### 4.4. The Americas

To date, no peer-reviewed studies from the Americas have reported detection of human-infecting HEV genotypes (HEV-1 to HEV-4) in shellfish. The only HEV-related detection comes from Brazil, where rat HEV (genus *Rocahepevirus*, species *Rocahepevirus ratti*; formerly HEV-C1) [18] was identified in mangrove-associated mussels and oysters at 2.2% (2/89), suggesting environmental circulation of non-human HEV variants rather than a foodborne human risk [87]. Other Brazilian studies have detected HAV and human astrovirus in commercial shellfish but not HEV RNA [92,93]. In Mexico, despite extensive research on HEV in humans, swine, and environmental sources, no HEV RNA has been reported in shellfish [94], underscoring a general lack of evidence for shellfish-associated HEV in North and South America.

### 4.5. Interpretation of Surveillance Data and Methodological Considerations

Taken together, global surveillance studies demonstrate that HEV RNA can be detected in shellfish across multiple regions and species, but prevalence and viral loads vary widely. These differences likely reflect environmental contamination levels, proximity to sewage or livestock effluent, species-specific feeding behavior, seasonal factors, and methodological variability.

Crucially, nearly all studies rely on molecular detection of HEV RNA, which does not indicate viral infectivity. Increasing adoption of standardized protocols—especially ISO/TS 15216-derived methods [88]—has improved comparability. However, heterogeneity persists in target tissues, extraction controls, detection limits, and reporting units (genome copies vs. international units), complicating cross-study comparisons. These issues are central when evaluating the public health implications of HEV RNA detection in shellfish and highlight the need for careful interpretation and further methodological harmonization.

## 5. Preventive Measures Against Shellfish-Associated HEV Infection

Although shellfish have not been definitively established as a confirmed vehicle for HEV transmission, the combined epidemiological and environmental evidence supports a precautionary approach, particularly for individuals at heightened risk for severe disease. Preventive strategies can be conceptualized at three complementary levels: (i) individual-level risk communication, (ii) public health guidance targeting vulnerable groups, and (iii) food preparation practices, especially thermal inactivation.

### 5.1. Risk-Based Prevention and Vulnerable Populations

In industrialized countries, infections with HEV-3/4 are generally self-limiting in immunocompetent individuals. However, certain groups face substantially increased risk of severe or chronic disease. These include solid-organ transplant recipients, patients receiving chemotherapy or immunosuppressive therapy, individuals with hematologic malignancies, and those with advanced HIV infection. Additionally, HEV infection during pregnancy—especially with HEV-1—has been associated with fulminant hepatitis and high maternal mortality in endemic settings [5,6,7].

Current clinical and public health recommendations for high-risk individuals already emphasize avoiding raw or undercooked pork, game meat, and liver products [95,96]. Given that shellfish can harbor HEV RNA in some geographic settings, raw or lightly cooked shellfish should be considered a potential—though not conclusively proven—source of exposure. Precautionary avoidance may therefore be prudent for vulnerable individuals. This aligns with established guidance for other enteric viruses and reflects a risk-management framework grounded in biological plausibility rather than confirmed causality [97].

### 5.2. Public Health Messaging and Clinical Awareness

From a public health standpoint, shellfish consumption represents a modifiable risk factor that can be addressed through targeted communication. Clinicians should remain aware that shellfish may appear in dietary histories of patients with acute hepatitis E, particularly in regions with documented HEV RNA detection in shellfish or where coastal waters are susceptible to contamination from sewage or livestock effluents. Although shellfish should not overshadow well-established HEV vehicles such as pork products, their inclusion in exposure assessments can enhance the completeness of epidemiological investigations [36].

Public health agencies may consider incorporating guidance on shellfish within broader HEV prevention materials, particularly for immunocompromised individuals and pregnant women. Such messaging should clearly convey existing uncertainties, emphasizing precaution without overstating risk, and situating shellfish within the broader context of foodborne and environmental exposure pathways.

### 5.3. Thermal Inactivation and Food Preparation Practices

Thermal treatment remains the most reliable method for inactivating HEV in food. Experimental studies indicate that HEV displays notable heat resistance, and insufficient cooking may leave infectious virus intact. Although most evidence derives from studies using HEV-contaminated meat matrices, heating food to an internal temperature of ≥71 °C for at least 20 min appears sufficient for complete inactivation [98,99].

Specific data on HEV in shellfish matrices remain limited. Nonetheless, given HEV’s demonstrated heat resistance and the well-established thermal sensitivity of other shellfish-borne viruses such as HAV [100,101], thorough cooking of shellfish—until the flesh becomes firm and opaque—should be recommended, especially for high-risk individuals. Common preparation methods involving brief steaming or cooking only until shells open may be insufficient to achieve uniform heat penetration and should not be relied upon for preventing HEV transmission [102].

### 5.4. Integration with Existing Food Safety Frameworks

At the population level, preventive strategies for shellfish-associated HEV exposure should be integrated into existing food safety and environmental monitoring systems. Current regulatory standards for shellfish harvesting primarily rely on bacterial indicators, which do not reliably reflect viral contamination. Expanded environmental surveillance—including monitoring of HEV in surface waters and shellfish-growing areas—may help identify higher-risk locations and inform targeted interventions, although such measures remain precautionary in the absence of confirmed shellfish-borne transmission [21].

In summary, preventive measures for potential shellfish-associated HEV infection should focus on protecting vulnerable populations through targeted dietary advice, clinician awareness, and promotion of adequate cooking practices. Until stronger causal evidence emerges, these measures represent proportionate, risk-based responses to a biologically plausible but incompletely defined exposure pathway.

## 6. Knowledge Gaps and Future Perspectives

Despite increasing epidemiological interest and accumulating surveillance data, substantial knowledge gaps remain regarding the potential role of shellfish in HEV transmission. Among these, the absence of direct infectivity evidence and the lack of molecular epidemiological linkage between shellfish-derived HEV and human infections represent the most significant barriers to establishing a causal relationship.

### 6.1. Lack of Direct Evidence for Infectivity of Shellfish-Associated HEV

Most studies investigating HEV in shellfish rely solely on detection of viral RNA. Although such findings demonstrate environmental contamination, they do not establish whether the detected genomes represent viable, infectious virions capable of initiating human infection. This limitation is critical because, for several enteric viruses, RNA can persist long after infectivity has been lost under certain environmental conditions [59,103].

For HAV and norovirus, shellfish-associated transmission has been established through a combination of outbreak investigations, infectivity assays, and, in some cases, human challenge studies or animal models [27,29,30,104]. In contrast, comparable infectivity data for HEV are scarce. Although substantial progress has been made in developing HEV cell culture systems—including those capable of supporting replication of HEV-3 and HEV-4 strains—these models have not yet been widely applied to shellfish-derived samples [105,106,107,108].

Technical challenges impede these efforts. Viral loads in shellfish are often low, frequently at or below quantification limits, hindering recovery of viable virus. Moreover, shellfish tissues contain substances that inhibit viral replication or interfere with cell culture assays. Developing reliable protocols for virus extraction, concentration, and infectivity assessment from shellfish matrices therefore remains a key research priority.

### 6.2. Absence of Molecular Epidemiological Linkage Between Shellfish and Human Infections

A second major knowledge gap concerns the lack of molecular epidemiological evidence directly linking HEV strains detected in shellfish to those recovered from human cases. For other foodborne viral pathogens such as HAV, outbreaks associated with shellfish have been confirmed by phylogenetic matching of patient-derived strains with viruses detected in implicated food items [27,29,30,104]. To date, no study has demonstrated such genetic identity—or close phylogenetic clustering—between HEV detected in shellfish and HEV isolated from epidemiologically linked human infections.

Several factors contribute to this gap:

- Limited availability of food samples during outbreaks. Shellfish samples are seldom collected at the time of exposure, and implicated items are typically unavailable once cases are identified.
- Short or partial viral sequences. Many studies generate only partial HEV sequences, insufficient for high-resolution phylogenetic analysis.
- High background diversity of HEV-3. The widespread circulation of closely related HEV-3 strains in both humans and animals complicates source attribution without near-complete genome sequences [5,109].

Future studies would benefit from integrated investigation frameworks combining systematic food sampling, comprehensive exposure assessments, and high-throughput sequencing approaches. Such designs could help distinguish coincidental co-circulation from genuine foodborne transmission events.

### 6.3. Methodological Standardization and Interpretation of Surveillance Data

Interpretation of surveillance data is further complicated by methodological heterogeneity. Variations in target tissues, virus recovery procedures, extraction controls, PCR targets, and reporting units all limit cross-study comparability. Although increasing use of ISO/TS 15216-based protocols has advanced standardization for enteric virus testing in shellfish, these methods were not specifically developed for HEV and may not optimally capture virus-specific features relevant to infectivity or risk assessment [88].

Furthermore, the relationship between HEV RNA levels in shellfish and actual infection risk remains undefined. Unlike norovirus and HAV—where dose–response relationships and outbreak-based exposure data exist—no comparable quantitative data are available for HEV. Without infectivity data or quantitative microbial risk assessment (QMRA) models, interpreting low-level HEV RNA detection in terms of public health risk remains challenging.

### 6.4. Future Research Priorities

Addressing these gaps will require multidisciplinary approaches integrating virology, environmental science, food safety, and epidemiology. Key research priorities include:

- Developing and validating methods to assess infectivity of HEV extracted from shellfish matrices.
- Applying whole-genome sequencing to both human and shellfish-derived HEV strains within coordinated surveillance systems.
- Improving understanding of environmental loading, persistence, and accumulation dynamics of zoonotic HEV genotypes.
- Integrating these data into quantitative microbial risk assessment models to evaluate public health relevance.

Progress in these areas is essential for moving beyond association-based inference toward a clearer determination of whether, and under what circumstances, shellfish constitute a meaningful source of HEV infection.

## 7. Conclusions

Accumulating epidemiological observations and environmental surveillance findings have increasingly drawn attention to shellfish as a potential source of HEV exposure. Reports of human HEV infection temporally associated with shellfish consumption, coupled with repeated detection of HEV RNA in bivalve mollusks across diverse geographic regions, support the biological plausibility of shellfish-mediated exposure. However, the current evidence base remains largely associative. In contrast to HAV and norovirus—where shellfish-borne transmission is firmly established [29,30]—there is still no direct demonstration that shellfish contain infectious HEV capable of initiating human infection, nor any definitive molecular epidemiological linkage between shellfish-derived HEV strains and human cases. As such, shellfish cannot presently be regarded as a confirmed vehicle of HEV transmission.

Despite these uncertainties, existing data have important implications for clinical management and public health. Given the potential severity of HEV infection in vulnerable populations, including immunocompromised individuals and pregnant women [11,12,110], a precautionary approach that considers shellfish among possible exposure routes is reasonable in appropriate settings. From a public health perspective, detection of HEV RNA in shellfish should be interpreted primarily as an indicator of environmental contamination rather than direct evidence of foodborne transmission. Nonetheless, these findings highlight the need for integrated surveillance encompassing aquatic environments, livestock sources, and food products.

Clarifying the role of shellfish in HEV transmission will require coordinated research efforts aimed at demonstrating infectivity of shellfish-derived HEV, establishing molecular epidemiological links between environmental and clinical strains, and harmonizing surveillance and methodological approaches. Until such evidence emerges, shellfish should be regarded as a plausible but unconfirmed component within the broader ecological network of HEV transmission.

## Figures and Tables

**Table 1:** Human hepatitis E cases and epidemiological studies in which shellfish consumption was implicated.

Region	Country/Setting	Study Type (Year)/Reference	Population/Cases	Shellfish Exposure Reported	Raw/Under-cooked Specified	Main Finding	HEV Genotype/Molecular Information
Europe	International cruise ship	Outbreak investigation (2009) [35]	789 passengers investigated; 33 acute HEV infections	Shellfish consumption on board	Not specified	Shellfish consumption significantly associated with acute HEV infection (OR 4.27; 95% CI 1.23–26.94)	HEV-3 detected in 3 cases; closely related to European strains
Europe	France (metropolitan, autochthonous cases)	National survey (2008) [36]	52 acute HEV cases; 47 indigenous	Uncooked shellfish consumption during incubation period	Yes (uncooked)	Uncooked shellfish listed among the most relevant/frequent reported risk factors	Genotype not central to analysis
Europe	Netherlands	Case–control study (2019) [37]	376 acute HEV cases; 1534 controls	Seafood/shellfish consumption (questionnaire-based)	Not specified	Shellfish not identified as a significant risk factor; pork products predominated	HEV-3 dominant among cases
Asia	Vietnam (travel-associated, Japan)	Case report (2004) [38]	Single imported case	Ingestion of uncooked shellfish during travel	Yes (uncooked)	Acute hepatitis E temporally associated with uncooked shellfish ingestion	HEV-4; 98.8% nucleotide identity to a Vietnamese isolate
Europe	France (southern regions)	Seroprevalence study + questionnaire (2015) [39]	3353 blood donors	Mussel consumption habit	Not specified	Anti-HEV IgG seropositivity associated with mussel consumption (*p* = 0.02)	Serology-based; reflects past exposure
Europe	France (nationwide)	Seroprevalence study + questionnaire (2016) [40]	10,569 blood donors	Oyster consumption	Not specified	Anti-HEV IgG associated with oyster consumption in multivariable analyses	Serology-based; nationwide dataset

HEV, hepatitis E virus; OR, odds ratio; CI, confidence interval; IgG, immunoglobulin G.

**Table 2:** Detection of hepatitis E virus (HEV) RNA in shellfish: country- and species-specific surveillance studies.

Region	Country	Study (Year)/Setting [Reference]	Shellfish Species (Main)	HEV RNA Positivity	Viral Load (If Available)	Genotype/Molecular Information
Europe	UK (Scotland)	Crossan et al. (2012)/intertidal sampling sites [31]	Mussels (*Mytilus* spp.)	85% (41/48)	4.25 (3.73–5.2) log_10_ IU/mL	HEV-3; high local contamination near slaughterhouse-influenced sites
Europe	UK (Scotland)	O’Hara et al. (2018)/retail (supermarkets) [32]	Mixed retail shellfish (mussels, oysters)	2.9% (9/310)	Low-level detection by RT-qPCR	HEV-3; retail-level contamination demonstrated
Europe	Spain (Galicia)	Rivadulla et al. (2019)/production and harvesting areas [33]	Mussels, clams, cockles	24.4% (41/168)	<10^2^–1.1 × 10^5^ copies/g digestive tissue	HEV-3 (subtype e–like); close relation to swine strains
Europe	Spain (Galicia)	Mesquita et al. (2016)/mussel batches [72]	Mussels (digestive tissue)	14.8% (12/81)	6.7 × 10^1^–8.6 × 10^4^ copies/g	HEV-3 (subtype e); batch-level quantification
Europe	Italy (southern regions)	La Rosa et al. (2018)/production areas [73]	Mussels and other bivalves	2.6% (10/384)	Not reported	HEV-3; environmental and shellfish samples
Europe	Italy (Apulia)	La Bella et al. (2021)/retail [74]	Mixed shellfish	0.9% (2/225)	2.5 × 10^2^, 7.0 × 10^1^ copies/g	Low-level retail contamination
Europe	Italy (Tuscany)	Donia et al. (2012)/four polluted sites [75]	Mussels	8.1% (3/37)	Not reported	HEV-1; HEV detected alongside other enteric viruses
Europe	Italy (Gulf of Naples)	Fusco et al. (2019)/production sites [76]	Mussels and clams	0% (0/289)	Not applicable	HAV detected (8.9%)
Europe	Denmark	Krog et al. (2014)/production areas [77]	Mussels	0% (0/29)	Not applicable	No HEV RNA detected
Europe	France	Grodzki et al. (2014)/environmental impact sites [70]	Oysters, mussels, clams	0% (0/286)	Not applicable	No HEV RNA detected
Europe	Germany	Rastar-Tangeten et al. (2025)/retail [78]	Mussels	0% (0/40)	Not applicable	No HEV RNA detected
Europe	Finland and Spain	Diez-Valcarce et al. (2012)/retail survey [79]	Mussels	3% (3/102)	127–348 copies/g (estimated)	HEV detected only in Spain (3/51) alongside other enteric viruses
Asia	China	Gao et al. (2015)/coastal and estuarine areas [34]	Ark shells, blood clams, Manila clams	17.5% (22/126)	Not reported	HEV-4; consistent with regional human/pig strains
Asia	China	Wei et al. (2025)/farmers’ markets in Hebei Province [80]	Bivalve shellfish	5.0% (12/240)	3312–20,350/2g	HAV detected (4/240)
Asia	China	Wang et al. (2025)/several markets in Hebei Province [81]	Bivalve shellfish	9.6% (34/354)	Not reported	HEV detected alongside other enteric viruses
Asia	South Korea	Song et al. (2010)/coastal regions [82]	Oysters	8.7% (14/161)	Not reported	HEV-3(3a); consistent with regional swine strains
Asia	Japan	Li et al. (2007)/river-associated packages [83]	Freshwater clams (*Corbicula japonica*)	6.3% (2/32)	Not reported	HEV-3; indicative of freshwater contamination
Asia	Japan	Ishida et al. (2012)/collected in Hokkaido [84]	Oysters	0% (0/517)	Not applicable	HEV detected in seawater (1/37)
Asia	Thailand	Namsai et al. (2011)/culture farm and retail markets [85]	Oysters, cockles, and mussels	0% (0/213)	Not applicable	HAV detected (8/213)
Africa	Morocco	Bazir et al. (2022)/coastal regions in Qualidia lagoo [86]	Oysters, clams	0% (0/104)	Not applicable	No HEV RNA detected
The Americas	Brazil	Figueiredo et al. (2025)/for local sale in a touristic area of Maranhao state [87]	Mangrove bivalve mollusks	2.2% (2/89)	Not reported	HEV-C1 (rat HEV)

Sample unit varies by study and may represent individual shellfish, pooled individuals, batches, or packages, as defined in the original publication. Therefore, direct comparison of positivity rates across studies should be interpreted with caution. Most studies analyzed digestive tissues (digestive gland) and used RT-PCR, RT-qPCR or RT-ddPCR-based methods, and methodological heterogeneity remains substantial. Detection of HEV RNA does not imply the presence of infectious virus, and no study to date has demonstrated direct molecular epidemiological linkage between shellfish-derived HEV and human cases. HEV, hepatitis E virus, RT-PCR: reverse transcription polymerase chain reaction; RT-qPCR: quantitative reverse transcription PCR; RT-ddPCR, reverse transcription droplet digital PCR; HAV, hepatitis A virus.

## Data Availability

All data are presented in the manuscript.
